# Identification and Analysis of Novel Inhibitors against NS3 Helicase and NS5B RNA-Dependent RNA Polymerase from Hepatitis C Virus 1b (Con1)

**DOI:** 10.3389/fmicb.2017.02153

**Published:** 2017-11-02

**Authors:** Na Yang, Chaomin Sun, Lixin Zhang, Jianguo Liu, Fuhang Song

**Affiliations:** ^1^Key Laboratory of Experimental Marine Biology, Institute of Oceanology, Chinese Academy of Sciences, Qingdao, China; ^2^Laboratory for Marine Biology and Biotechnology, Qingdao National Laboratory for Marine Science and Technology, Qingdao, China; ^3^State Key Laboratory of Bioreactor Engineering, East China University of Science and Technology, Shanghai, China; ^4^Key Laboratory of Pathogenic Microbiology and Immunology, Institute of Microbiology, Chinese Academy of Sciences, Beijing, China

**Keywords:** hepatitis C virus, NS3 helicase, NS5B RNA-dependent RNA polymerase, high throughput, inhibitor

## Abstract

Hepatitis C virus (HCV) leads to severe liver diseases, including liver fibrosis, cirrhosis and hepatocellular carcinoma. Non-structural protein 3 helicase (NS3h) and non-structural protein 5B RNA-dependent RNA polymerase (NS5B) are involved in the replication of HCV RNA genome, and have been proved to be excellent targets for discovery of direct-acting antivirals. In this study, two high-throughput screening systems, fluorescence polarization (FP)-based ssDNA binding assay and fluorescence intensity (FI)-based dsRNA formation assay, were constructed to identify candidate NS3h and NS5B inhibitors, respectively. A library of approximately 800 small molecules and crude extracts, derived from marine microorganisms or purchased from the National Compound Resource Center, China, were screened, with three hits selected for further study. Natural compound No.3A5, isolated from marine fungi, inhibited NS3h activity with an IC_50_ value of 2.8 μM. We further demonstrated that compound No.3A5 inhibited the abilities of NS3h to bind ssDNA in electrophoretic mobility shift assay and to hydrolyze ATP. The NS3h-inhibitory activity of compound No.3A5 was reversible in our dilution assay, which indicated there was no stable NS3h-No.3A5 complex formed. Additionally, compound No.3A5 exhibited no binding selectivity on NS3h or single strand binding protein of *Escherichia coli*. In NS5B assays, commercial compounds No.39 and No.94 previously reported as kinase inhibitors were found to disrupt dsRNA formation, and their IC_50_ values were 62.9 and 18.8 μM, respectively. These results highlight how identifying new uses for existing drugs is an effective method for discovering novel HCV inhibitors. To our knowledge, all inhibitors reported in this study were originally discovered with HCV anti-non-structural protein activities *in vitro*.

## Introduction

Hepatitis C virus (HCV), which leads to liver fibrosis, steatosis, cirrhosis and hepatocellular carcinoma, is a serious health problem affecting about 200 million people worldwide (Webster et al., 2009; Shi et al., 2015). In China, the most widely used anti-HCV therapy is a combination of pegylated interferon alpha and ribavirin, which causes severe side effects and has a low success rate (Rao et al., 2017). Significantly, no preventative vaccines against HCV are currently available. Thus, urgent work for the discovery of new classes of antiviral compounds has been widely undertaken.

The genome of HCV is a single positive-stranded RNA and encodes a long polyprotein that contains structural and non-structural proteins, including Core, Envelope 1 (E1), E2, P7, Non-structural Protein 2 (NS2), NS3, NS4A, NS4B, NS5A and NS5B (Han et al., 2009). In recent years, a better understanding of the HCV life cycle has led to the development of direct antiviral agents (DAA) that target non-structural proteins. In 2011, telaprevir and boceprevir targeting NS3/4A were approved as first-generation DAA drugs (Yu et al., 2014). Certainly, non-structural proteins NS3h and NS5B, which are essential for viral replication (Suzuki et al., 2007), can also be developed as targets for developing direct anti-HCV drugs. NS3h can unwind dsRNA, dsDNA and RNA/DNA heteroduplexes in a 3′-5′ direction using a nucleoside triphosphate as the energy source (Furuta et al., 2014). Additionally, NS5B encodes an RNA-dependent RNA polymerase, which catalyzes the formation of dsRNA from a ssRNA template both *in vivo* and *in vitro* (Yamashita et al., 1998). To date, sofosbuvir and dasabuvir have been approved for the treatment of hepatitis C infection by deactivating NS5B (Cholongitas and Papatheodoridis, 2014; Gentile et al., 2014). Combination treatments with a protease inhibitor (e.g., simeprevir) and a NS5B inhibitor have achieved a very high sustained virological response in HCV patients (Choop et al., 2015). However, the frequency of reports describing the appearance of drug-resistant HCV variants dramatically increased during DAA monotherapy (Bacon et al., 2011; Sherman, 2011). In this light, the discovery of novel HCV inhibitors still attracts the attention of researchers.

Naturally occurring products are an important source of structurally diverse and biologically active secondary metabolites. The diversity of organisms in the marine environment has provided new drugs in almost all therapeutic areas. To date, several clinical drugs derived from the marine environment are used as anticancer, antiviral, pain control, and hypertriglyceridemia agents (Gerwick and Moore, 2012). Active agents, such as crude extracts from sponge *Amphimedon* sp. and *Alloeocomatella polycladia*, pure compounds suvanine, hydroxyanthraquinones and manoalide *etc*. were all reported as novel NS3h inhibitors of HCV (Salam et al., 2012; Yamashita et al., 2012; Furuta et al., 2015). In order to discover novel antiviral drugs against HCV, small molecular compounds derived from marine microorganisms were screened. On the other hand, identifying new uses for existing drugs is also one of the solutions to combat rapidly emerging disease. A considerable amount of time and money can be saved by using existing drugs with known pharmacokinetics and safety profiles during drug discovery (Chong and Sullivan, 2007). For instance, anti-fungal agent itraconazole also demonstrated anti-cancer and anti-enteroviral activities by targeting oxysterol-binding protein and OSBP-related protein 4 (Strating et al., 2015), while the immunosuppressive agent rapamycin was found to inhibit pancreatic tumor growth (Morran et al., 2014). Hence, commercial compounds used for controlling hepatoma cell proliferation purchased from National Compound Resource Center, China were also applied to anti-HCV screening in this study.

In this study, two high-throughput screening systems, FP-based ssDNA binding assay and FI-based dsRNA formation assay, were constructed to identify candidate NS3h and NS5B inhibitors, respectively. One natural compound, No.3A5, was found to inhibit NS3h binding to ssDNA, and was further investigated for its inhibition mechanism. On the other hand, compounds No.39 and No.94, two kinase inhibitors, were discovered to disrupt dsRNA formation. To our knowledge, all inhibitors were originally reported with anti-HCV activities *in vitro*.

## Materials and Methods

### Compounds

Small molecular compounds and crude extracts derived from marine microorganisms were all obtained from Prof. Lixin Zhang’s lab, East China University of Science and Technology, Shanghai, China. Commercial compounds were purchased from National Compound Resource Center, China (**Supplementary Data Sheets 1**, **2**).

### Expression and Purification of NS3h and Truncated NS5B

Recombinant NS3h was expressed and purified as described below. cDNA genome of HCV 1b (Con1) was kindly provided by Prof. Charles M. Rice, the Rockefeller University, New York. NS3h (468 amino acids, corresponding sequence of pH77 genome GenBank No. AJ238799.1: 3918 to 5321) was expressed and purified in *Escherichia coli* BL21 (DE3) cells as recombinant proteins. The *ns3h* gene of HCV 1b (Con1) was cloned into pET21b(+) vector (Novagen). After sequencing, the right construct was transformed into *E. coli* BL21 (DE3) cells. Cells harboring plasmids were grown to an absorbance at 600 nm (*A*_600_) of 0.6 and induced with 0.5 mM isopropyl-D-thiogalactopyranoside at 16°C overnight. Cells were harvested by centrifugation, washed in HEPES buffer (25 mM, pH 7.4) and resuspended in buffer A containing 25 mM HEPES (pH 7.4), 75 mM KCl, 10% glycerol, 1 mM DTT and 0.5 % protease inhibitor cocktail (Sigma–Aldrich). For purification, cells were lysed by sonication and centrifuged at 25,000 × *g*. The supernatant was then applied to a 5 mL HisTrap FF column (GE Healthcare) and equilibrated in buffer A. Then the column was eluted by increasing imidazole concentration to 300 mM using ÄKTA protein purification system (GE Healthcare). The fraction containing NS3h was confirmed by SDS-PAGE. Then the NS3h fraction was dialyzed against buffer A and loaded on a HiTrap Q FF column (GE Healthcare) to exclude nucleic acids contamination. The column was eluted by increasing KCl concentration to 500 mM. After SDS-PAGE confirmation, fractions containing NS3h were dialyzed again using buffer A, and then was concentrated and loaded onto HiLoad 16/600 Superdex 200 (GE healthcare) and eluted by buffer A for further purification. The purified NS3h was stored at -80°C. Protein concentration was quantified via spectrophotometry.

Plasmid pET11a::*ns5b* for expressing truncated NS5B (hydrophobic 21 amino acids were deleted at C terminal) of HCV 1b (Con1) was kindly provided by Prof. Helena Danielson, Uppsala University, Sweden (Abdurakhmanov, 2010). Recombinant protein NS5B was expressed, purified, stored and quantified as NS3h.

### Fluorescence Polarization (FP) Based ssDNA Binding Assay

FP assays were performed in 96-well, flat bottom, black plates and the final volume of each reaction was 50 μL. The reaction system contained 5 nM Cy5-dT15 (synthesized by Sangon Biotech, China), 15 nM NS3h, 25 mM MOPS (pH 7.5), 1.25 mM MgCl_2_, 0.0025 mg/mL BSA, 0.005 % (v/v) Tween 20 and 0.025 mM DTT. 0.5 μL compound dissolved in DMSO with a final concentration of 100 μM or 100 μg/mL was added. For each plate, blank wells (without NS3h and Cy5-dT15) and reference blank wells (without NS3h) were included. Positive control and negative control were primuline (Li et al., 2012) and DMSO, respectively. FP value was detected using a TECAN Infinite M1000 Pro multi-mode microplate reader, with detection wavelength of excitation 635 nm. Inhibition value was calculated as (mp_sample_-mp_negative_)/(mp_positive_-mp_negative_) × 100% (mp standards for polarization value).

### Fluorescence Intensity (FI) Based dsRNA Formation Assay

Anti-NS5B assay was performed in 25 μL reaction system as reported (Eltahla et al., 2013). The reaction system contained 250 μM rGTP, 10 μg/mL poly(C) RNA (Sigma–Aldrich, p4903), 2.5 mM MnCl_2_, 5 mM DTT, 0.01% BSA, and 0.005 % Tween-20 in 20 mM Tris-Cl (pH 7.5). 0.2 μL DMSO or compound dissolved in DMSO with a final concentration of 100 μM or 100 μg/mL was added to each well. Heat-inactivated NS5B was used as positive control. Plates were incubated at 30°C for 1 h. Then 175 μL PicoGreen dye (1:350 diluted in TE buffer) was added to a final volume of 200 μL. After covering from light for 5 min, FI was measured using a TECAN Infinite M1000 Pro multi-mode microplate reader at excitation wavelength of 485 nm and emission wavelength of 520 nm.

### Validation of High-Throughput Screening (HTS) Assays

The Z′ factor and coefficient of variation (CV) values were used to evaluate the quality of HTS systems and were calculated as follows:

Z′ = 1-(3SD of positive control + 3SD of negative control)/| mean of positive control - mean of negative control|, SD: Standard deviation. The theoretical value is between 0.5 and 1 (Sui and Wu, 2007). CV(%) = SD_max_/Mean_max_ or CV(%) = SD_min_/Mean_min_. The acceptable value of CV for HTS assay is less than 10%.

### Electrophoretic Mobility Shift Assay (EMSA)

Whether purified NS3 helicase can bind to DNA substrate *in vitro* was tested using EMSA. The binding assays containing 50 mM Tris-Cl (pH 7.4), 10% glycerol, 100 nM DNA substrate (5′-Cy5-CC TAC GCC ACC AGC TCC GTA GG–3′ annealed to 5′-GGA GCT GGT GGC GTA GG (T)20-3′) (Mukherjee et al., 2012) and 650 nM NS3h were incubated at 0°C for 20 min. Aurintricarboxylic acid was used as positive control (Shadrick et al., 2013). A non-denaturated PAGE gel was pre-run at 4 °C for 30 min at 120 V. Samples were loaded and ran 40 min at 120 V. Then the resulting gel was visualized by fluorescence imaging using Typhoon FLA 9500 with the wavelength of 635 nm.

### ATPase Assay

ATPase assays were assembled in 96-well, flat bottom, transparent plates on a TECAN Infinite M1000 Pro multi-mode microplate reader, with an absorbance at 630 nm, and the total reaction volume of each well was 50 μL. The final reaction system contained 15 nM NS3h, 1 mM ATP, 25 mM MOPS (pH 6.5), 1.25 mM MgCl_2_, 5% DMSO, 50 μg/mL BSA and 0.01 % Tween 20. Plates were incubated at 23°C for 15 min. Then 200 μL malachite green solution (0.034% malachite green, 1 M HCl, 1% ammonium molybdate, and 0.025% Tween 20 followed within 10 s by addition of 25 μL of 35% sodium citrate) was added to each well for 20 min at 23°C to terminate the reaction.

### *Escherichia coli* Single-Strand Binding (SSB) Protein Assay

Expression plasmid pET21b(+)::*ssb* was constructed and SSB protein was obtained as NS3h. The procedure for this assay was the same as that for NS3h except that *E. coli* SSB was used at 20 nM instead of NS3h.

## Results

### Establishment and Validation of FP Based ssDNA Binding Assay

**FIGURE 1:**
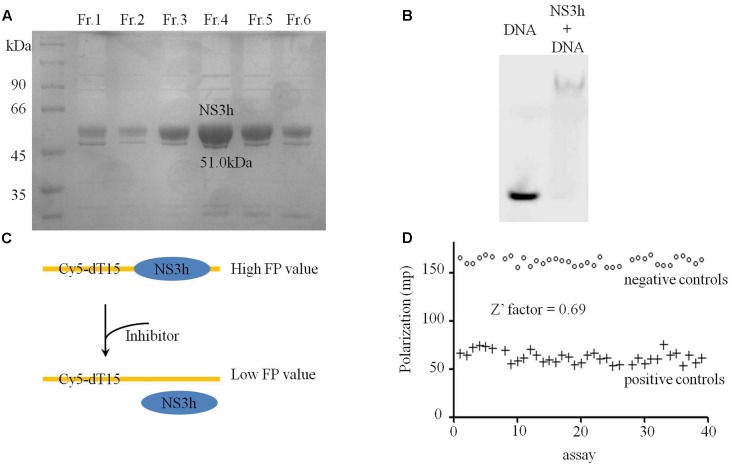
Purification and binding assays of NS3h with nucleic acids. **(A)** Coomassie stained SDS-PAGE gel analysis of purified NS3h. Molecular weight (MW) markers were shown in kilodaltons. **(B)** Native agarose gel showing binding of NS3h to nucleic acids. **(C)** The schematic diagram of FP based assay on the interaction between NS3h and ssDNA. **(D)** Fluorescence polarization of 40 positive control assays (100 mM primuline) and 40 negative control assays (DMSO).

Establishing effective HTS systems and identifying potential new HCV replication inhibitors were the primary objectives of this study. A FP-based ssDNA binding assay was selected for NS3h inhibitors discovery. NS3h of HCV 1b (Con1) was successfully expressed in *E. coli* BL21(DE3) cells and purified by Ni-NTA column chromatography, Q column chromatography and gel filtration column chromatography. NS3h has a molecular mass of 51.0 kDa (**Figure 1A**) and the total yield was 0.75 mg/L LB broth. The *in vitro* binding capacity of NS3h with nucleic acids was verified by EMSA, which indicated that purified NS3h could tightly bind to the oligo-dT sequence of dsDNA and that the NS3h-dsDNA complex migrated much more slowly than free dsDNA through a non-denaturing polyacrylamide gel (**Figure 1B**).

After optimization, the reaction conditions for this assay were 10–20 nM NS3h, 5 nM Cy5-dT15, 25 mM MOPS (pH 7.5), 0–1.5 mM MgCl_2_, 0.0025 mg/mL BSA, 0.005 % (v/v) Tween 20 and 0.025 mM DTT. There were significant differences in FP values for DMSO concentration ranges of 0–40% (v/v) and the best range for this assay was 0–5%. Decreased FP values were detected with increasing concentrations of the positive control (primuline) or competitive substrate non-labeled dT20. FP-based binding assays were then performed in a high-throughput format to evaluate necessary precision and reproducibility. To determine well-to-well variation by FP-based binding assays, 40 negative controls (1% v/v of DMSO only) and 40 positive controls (100 μM primuline) were performed, and resulted in a Z′ factor of 0.69 (>0.5) (**Figures 1C,D**), which is considered acceptable for HTS system. The CV values of HTS system were CV_max_ = 2.6% and CV_min_ = 9.5%. Both values were less than the threshold value of 10%, which is recognized as delineation of correct assays.

### Compound No.3A5 Prevents NS3h from Binding ssDNA

**FIGURE 2:**
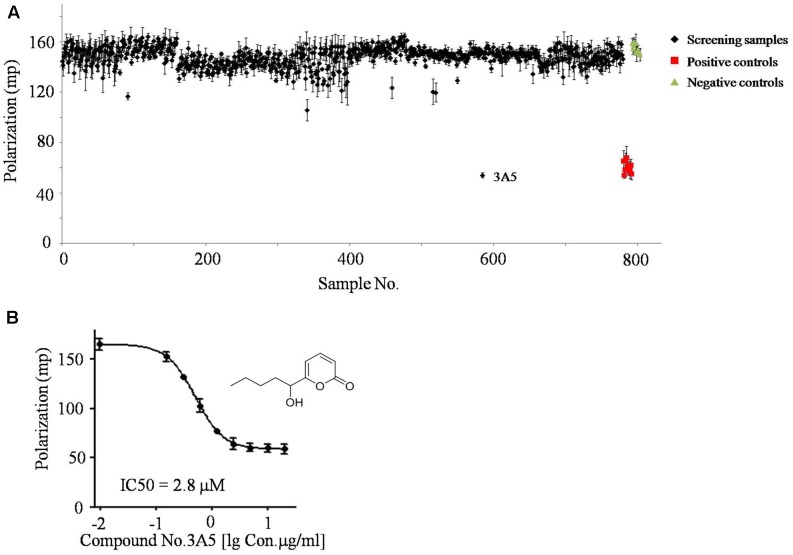
The screening results of FP based ssDNA binding assay. **(A)** About 800 small molecular compounds and crude extracts were screened using the FP system. **(B)** IC_50_ assay of compound No.3A5 toward the interaction between NS3h and ssDNA.

Sets of small molecular compounds and crude extracts were screened by the FP-based ssDNA binding assay in 96-well plates. Samples were added to a final concentration of 100 μM or 100 μg/mL to the reaction system (**Figure 2A** and **Supplementary Data Sheet 1**). In additional positive control assays, primuline was added to a final concentration of 100 μM (**Figure 2A**). Three parallel repeats were employed for the primary FP screening and positive hits were selected for activity confirmation. Natural compound No.3A5, isolated from marine fungi, exhibited significantly lower FP signals than other samples and inhibited the ability of NS3h to bind Cy5-dT15 in a concentration dependent manner. The IC_50_ value of compound No.3A5 was determined to be 2.8 μM (**Figure 2B**). Thus, compound No.3A5 was proposed to disrupt the interaction between NS3h and ssDNA and was selected for further study.

**FIGURE 3:**
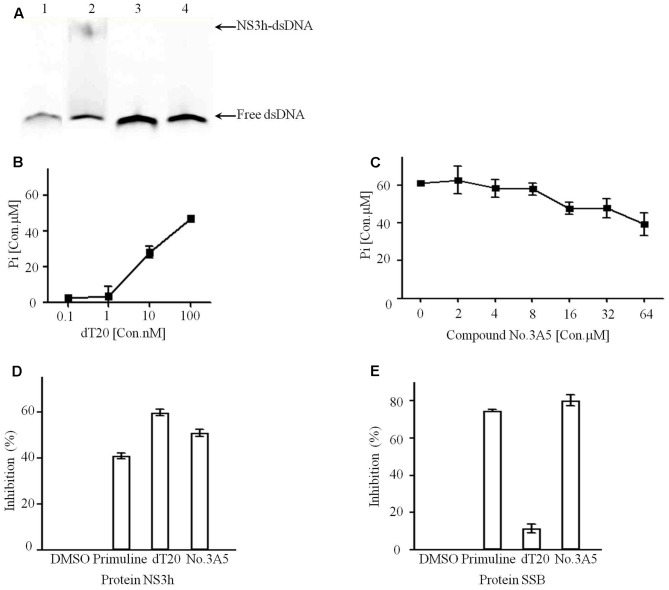
Active compound No.3A5 disrupt the interaction between NS3h and nucleic acids. **(A)** EMSA. Samples were examined on a 15% native polyacrylamide gel. Lane 1, Cy5-dT20 only; lane 2, Cy5-dT20, NS3h and DMSO; lane 3, Cy5-dT20, NS3h and 50 μM aurintricarboxylic acid; lane 4, Cy5-dT20, NS3h and 1 mM compound No.3A5. **(B)** In ATPase assays, addition of dT20 enhanced ATP hydrolyzation. **(C)** Increasing amount of compound No.3A5 could affect ATPase of NS3h. **(D)** Inhibition results showed compound No.3A5 had no binding selectivity on NS3h. **(E)** Inhibition results showed compound No.3A5 had no binding selectivity on SSB protein of *Escherichia coli*.

Compound No.3A5 has a molecular weight of 182.1 Da and has no previously reported biological activity. Therefore, EMSA assay was performed to further validate the effects of compound No.3A5 on the interaction of NS3h and nucleic acids. The reaction system included 50 mM Tris-Cl (pH 7.4), 10% glycerol, 100 nM DNA substrate and 650 nM NS3h. Notably, in the presence of compound No.3A5, no NS3h-dsDNA complex band was detected, which showed the same result with aurintricarboxylic acid, a known NS3h inhibitor (**Figure 3A**). Protein NS3h catalyzes ATP hydrolysis and dsDNA/RNA unwinding and the unwinding ability of NS3h is dependent on ATP binding and ATP hydrolysis (Frick, 2007). We explored the effects of compound No.3A5 on the ATPase activity of NS3h. Firstly, the ATP hydrolysis ability of purified NS3h was investigated and we found that increasing dT20 concentration enhanced ATP hydrolysis (**Figure 3B**). This phenomenon verified that binding capacity of NS3h with nucleic acids needs energy produced by ATP hydrolyzation. Then, compound No.3A5 was added to the ATPase reaction solution. As shown in **Figure 3C**, with increasing concentrations of compound No.3A5 from 0 to 64 μM, decreased concentrations of Pi were detected, suggesting that compound No.3A5 could affect the ATPase activity of NS3h. Next, the ability of compound No.3A5 to function as a reversible inhibitor was tested. We incubated 1 μM NS3h with 3 μM compound No.3A5 for 60 min and then diluted the protein 60-fold while adding it to reaction system to determine if the protein retained the ability to bind ssDNA with a similar affinity as the protein when it was treated with DMSO only. Interestingly, when NS3h was treated with 3 μM compound No.3A5, the protein retained the ability to bind ssDNA after dilution. This suggests that there was no formation of covalent bond between compound No.3A5 and NS3h and the inhibition was reversible. Finally, to test if compound No.3A5 inhibited NS3h specifically, the same FP-based assay was repeated, substituting NS3h with the *E. coli* single-stranded DNA binding (SSB) protein. Gene *ssb* from *E. coli* DH5α genome was cloned and SSB protein (MW: 19.0 kDa) was expressed and purified as NS3h. Control primuline inhibited the ability of SSB to bind Cy5-dT15. Similarly, compound No.3A5 also inhibited the ability of *E. coli* SSB to bind Cy5-dT15, consistent with the result that there was no covalent bond formation between compound No.3A5 and NS3h. In contrast, competitive substrate non-labeled dT20 showed high selectivity on NS3h (>60% inhibition) and SSB protein (<10% inhibition) (**Figures 3D,E**). So non-specific interaction of compound No.3A5 with ssDNA binding proteins was detected.

### FI Based dsRNA Formation Assay

**FIGURE 4:**
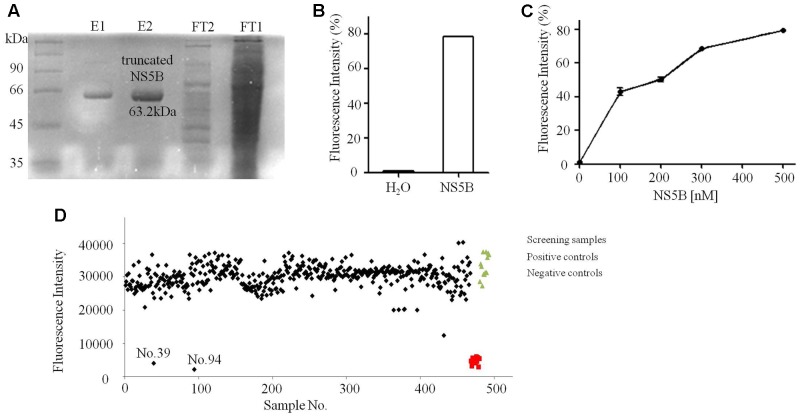
Purification and FI based dsRNA formation assays of truncated NS5B. **(A)** Coomassie stained SDS-PAGE gel analysis of purified truncated NS5B with 6× His tags. **(B)** Truncated NS5B catalyzed dsRNA formation (adding H_2_O or 300 nM truncated NS5B in reaction system). **(C)** The effect of truncated NS5B concentration on dsRNA formation measured using the PicoGreen. **(D)** About 800 small molecular compounds and crude extracts were screened using the FI system.

In this study, the FI-based dsRNA formation assay was selected for discovery of NS5B inhibitors. Truncated NS5B of strain 1b (Con1) (21 hydrophobic amino acids deletion at C terminal, MW: 63.2 kDa) was expressed and purified as described (**Figure 4A**). As reported by Yamashita et al. (1998), NS5B catalyzes the formation of dsRNA from ssRNA template *in vitro.* Combined with PicoGreen, a reagent that preferentially binds dsRNA compared with ssRNA, can be used to quantify dsRNA by detecting FI excited at wavelength 485 nm (Mastrangelo et al., 2012). The catalytic ability of purified truncated NS5B was confirmed, with a significant increase of dsRNA (**Figure 4B**). Meanwhile, with increasing concentrations of truncated NS5B, the FI was greatly enhanced (**Figure 4C**). Therefore, we sought to establish another HTS system based on the truncated NS5B [HCV 1b (Con1)] and ssRNA, and FI was used for dsRNA amount detection. Tolerance to BSA, DMSO and Tween-20 was tested to optimize concentrations for this HTS system. The final reaction solution in 96-well plate was considered as 230 μM rGTP, 10 μg/mL poly(C) RNA, 2.5 mM MnCl_2_, 5 mM DTT, 0–0.01% BSA, and 0–0.005 % Tween-20 in 20 mM Tris-Cl (pH 7.5), 0–4% DMSO. The final value of Z’ factor was 0.67.

### Compounds No.39 and No.94 Inhibit dsRNA Formation

**FIGURE 5:**
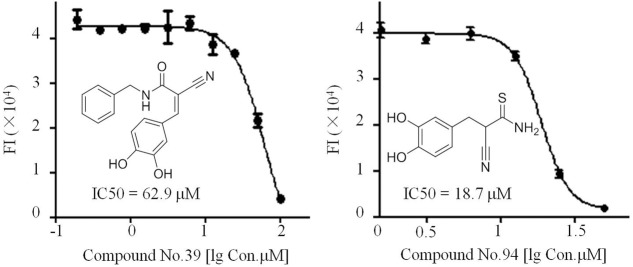
IC_50_ assays of compounds No.39 and No.94 toward the dsRNA formation catalyzed by truncated NS5B.

Of all the tested samples (**Figure 4D** and **Supplementary Data Sheet 2**), compounds No.39 and No.94 with low FI values were confirmed after primary and secondary screening. They both reacted in a dose-dependent manner and the IC_50_ values were determined to be 62.9 and 18.7 μM, respectively (**Figure 5**). These two compounds are both known as tyrosine kinase inhibitors. In this study, disrupting dsRNA formation is the novel function of them, which is worth further study.

## Discussion

The high prevalence of liver disease caused by HCV has stimulated the search for more effective and safer drugs (Trahtenherts et al., 2008). Current therapy combines pegylated interferon, ribavirin and newly developed DAA agents to treat HCV infected patients (McHutchison et al., 2009; Imamura and Chayama, 2015; Jacobson et al., 2016). However, due to various HCV genotypes and high mutation rates in the HCV genome, the discovery of novel HCV inhibitors still attracts researcher’s attention. Lots of antiviral campaigns for active compounds have been developed against HCV, mainly single enzyme based assay *in vitro* or HCV replicon based cell model *in vivo*. NS3h and NS5B are attractive targets for drug development given the crucial role that these enzymes play in the viral life cycle (Mukherjee et al., 2012; Yamashita et al., 2012). Since the majority of Chinese HCV infections are caused by HCV genotype 1b (Con1), this genotype was chosen for antiviral compounds screening in this study. We successfully established FP-based and FI-based high-throughput screening systems targeting NS3h and truncated NS5B of HCV 1b (Con1), respectively. Totally, a library of approximately 800 compounds and crude extracts derived from marine fungi, marine actinomycetes and ‘old’ synthetic compounds purchased from National Compound Resource Center, China were applied to these two HTS systems. Three compounds were confirmed as positive hits which demonstrated the effectiveness of our HTS systems.

It is difficult for small molecules, especially those of molecular weight < 500, to inhibit NS3h. Most HTS systems designed to identify inhibitors of NS3h-catalyzed DNA strand separation achieved few inhibitors (Mukherjee et al., 2014). These HCV NS3h inhibitors can act by inhibiting NTP binding, nucleic acid binding, ATP hydrolysis and unwinding of nucleic acids (Kwong et al., 2005). Elucidation of inhibitory mechanisms provides useful information on the utilization of these inhibitors. In our approach, small compound No.3A5 derived from marine fungi without any reported biological activity could disrupt the interaction between NS3h and ssDNA using FP based nucleic acid binding assay (**Figure 2B**). Combined with EMSA results, compound No.3A5 showed obvious inhibitory effect on NS3h-DNA complex formation, which arouses our interest for its mode of action. Besides inhibiting nucleic acid binding, compound No.3A5 had effect on the activity of the NS3h ATPase (**Figure 3C**). The ability of compound No.3A5 to inhibit HCV NS3h was reversible, which means there was no formation of covalent adducts between compound No.3A5 and NS3h. During a selective test, >80% *E. coli* single-stranded DNA binding proteins were deactivated by adding compound No.3A5. Hence, compound No.3A5 functioned non-specifically, which was consistent with known NS3h inhibitors such as mitoxantrone and primuline (Mukherjee et al., 2014) (**Figures 3D,E**). In addition to NS3h inhibitors, compounds No.39 and No.94 were found to disrupt the dsRNA formation catalyzed by NS5B. Compounds No.39 and No.94 have been studied for many years as tumor inhibitors by targeting tyrosine kinase, an EGF (epidermal growth factor) receptor (Rashid et al., 2015). In our observation, NS5B might be another target of compounds No.39 and No.94 (**Figure 5**). Obviously, both compounds have ortho-hydroxy benzene and cyanogen groups, which could help to guide the further chemical development of this scaffold. The structure-activity relationships between these analogs would need to be taken in such assays. Unfortunately, the active and selective properties of these compounds in cells bearing HCV replications were not performed, and also the relative toxicity of these compounds to hepatocytes. Hence, series studies *in vivo* are needed in future.

In summary, the HTS systems established in this study were effectively performed to discover new inhibitors for HCV 1b (Con1). Small molecular compounds No.3A5, No.39 and No.94 were identified and the action mechanism of compound No.3A5 was preliminary discussed in this study.

## Author Contributions

NY performed all of the experiments. NY and CS conceived and designed the experiments. LZ supplied all the tested small molecular compounds and polysaccharides. NY, FS, and JL analyzed the data, prepared the figures and wrote the paper.

## Conflict of Interest Statement

The authors declare that the research was conducted in the absence of any commercial or financial relationships that could be construed as a potential conflict of interest.

## Supplementary Material

**DATA SHEET 1 |** Screening results targeting NS3h.

**DATA SHEET 2 |** Screening results targeting truncated NS5B.
